# Disparity between capacity and performance in the International Classification of Functioning: implications for functionality in older adults

**DOI:** 10.3934/publichealth.2025040

**Published:** 2025-08-05

**Authors:** María Cristina Ruiz-Garrós, Ana Alejandra Laborda Soriano, Alba Cambra-Aliaga, Pilar Dominguez-Oliván, Marta Perez-de-Heredia-Torres, Laura Gonzalo-Ciria, Ana Gascón-Catalán

**Affiliations:** 1 Department of Physiatry and Nursing, Faculty of Health Sciences, University of Zaragoza, Zaragoza, Spain; 2 Department of Physical Therapy, Occupational Therapy, Rehabilitation and Physical Medicine, Faculty of Health Sciences, Rey Juan Carlos University, Madrid, Spain

**Keywords:** capacity, performance, functioning, activity limitations, occupational balance, participation restrictions

## Abstract

**Background:**

Functioning, recognized as the third health indicator and a key metric for rehabilitation, can be assessed by measuring capacity and performance.

**Objective:**

To quantify the prevalence of disability and cognitive impairment in individuals aged 50+ and evaluate the reliability and clinical relevance of capacity and performance qualifiers in the activity and participation domains of the international classification of functioning checklist (ICF checklist).

**Methods:**

A cross-sectional study was conducted in a population from rural and urban areas of Aragón (Spain), including 1707 participants. Disability and cognitive impairment were assessed using the WHO Disability Assessment Schedule 12-item version (WHODAS 12) and mini-mental state examination. A randomly selected subsample (n = 129) underwent a detailed functional evaluation. The ICF checklist was used to compare capacity and performance, analyzing their agreement and differences.

**Results:**

Disability was present in 50.6% of participants. Severe or total disability was most prevalent in general tasks/demands (10.1%) and domestic life (7.1%), reflecting significant daily functioning limitations. The largest capacity-performance discrepancies were in domestic life, self-care, and learning, indicating key intervention areas. Notably, 40.5% of participants had lower performance in domestic life, followed by learning/knowledge (28%) and mobility (17%). Conversely, performance exceeded capacity in community living (13.3%) and personal relationships (5.5%), highlighting the influence of environmental factors.

**Conclusions:**

Disability is highly prevalent in adults aged 50+, notably affecting daily functioning. Gaps between ability and performance indicate environmental barriers, especially at home and in learning contexts. Improved outcomes in social domains suggest enabling conditions. Findings support the ICF checklist's clinical value and advocate for integrating environmental factors into disability care.

## 1. Introduction

### 1.1. General considerations

The older population is particularly susceptible to chronic non-communicable diseases, which increase the risks of disability, hospitalization, institutionalization, and premature mortality [1]–[3]. In clinical practice, the assessment of physical health provides essential insights into the overall health status of older adults. Such evaluations are critical for gauging the effectiveness of health interventions, including rehabilitation, and for understanding the impact of specific events or conditions, such as hospitalization or the onset of chronic disease 4. In 2001, the World Health Organization (WHO) introduced the international classification of functioning, disability and health (ICF), establishing a standardized framework and shared terminology for describing health and its related components 5. Later, in 2013, the WHO published a manual detailing measurement instruments and standardized procedures to ensure reliable and meaningful assessments of functioning 6. Despite these advances, further research is required to clarify and interpret discrepancies between the ICF's capacity and performance qualifiers [7]–[12]. The ICF also identifies environmental facilitators and barriers that can be targeted for intervention and serves as a guide for interdisciplinary assessments 6. Furthermore, it enables the evaluation of the gap between actual and potential functioning, using capacity and performance qualifiers. This information helps health professionals assess the potential for performance improvement with appropriate support and facilitates the development of tailored intervention plans focused on domains with the highest potential for progress. These components have emerged as strong predictors of frailty in older adults and play a central role in assessing the need for care and support for individuals and their families [13]–[20]. Participation is increasingly recognized as a critical outcome of rehabilitation, prompting the development of assessment tools, such as the ICF checklist (ICF-C) and WHODAS 2.0. However, relatively few studies have investigated the content of these instruments to determine how participation is evaluated, emphasizing the need to assess the reliability of ICF codes and consider potential revisions to the ICF-C. This includes the development of condition-specific core sets that standardize the domains to be measured for each chronic condition. Currently, functioning may be assessed through a dual lens—capacity and performance—which offers a nuanced understanding of disability [19],[21]–[24]. The assessment of functioning using the WHO's ICF-C is especially relevant within the framework of integrated care for older adults, as outlined in the WHO's integrated care for older people (ICOPE) guidelines. ICOPE emphasizes the early detection of declines in intrinsic capacity across key domains, including mobility, cognition, nutrition, and sensory function. The ICF's standardized framework and common terminology support comprehensive functional evaluations and strengthen the implementation of proactive, person-centered care strategies aimed at maintaining functional ability in aging populations 25.

This study employed the ICF-C as a practical and standardized tool for assessing disability in individuals aged 50 and older. Its concise yet comprehensive format allows for efficient evaluation of core domains of functioning, supporting early identification of limitations and guiding timely public health interventions to prevent disability progression and promote healthy aging. While the ICF Geriatric Core Set offers a more detailed assessment for older adults with complex health conditions, its operational demands reduce its feasibility in population-based studies involving heterogeneous age groups starting from midlife. Many individuals in this age range have not yet developed advanced geriatric syndromes or significant functional decline, rendering the use of highly specialized tools potentially excessive and burdensome in terms of time, training, and data collection. In contrast, the ICF-C presents a more feasible and adaptable alternative for large-scale field research. Its broad applicability across diverse community settings makes it particularly suitable for functional assessment in aging populations within public health contexts 26.

According to the WHO's ICF framework, capacity refers to an individual's ability to execute a task or action in a standardized environment, reflecting what the individual is capable of under ideal conditions without contextual influences. In contrast, performance captures the actual execution of tasks or actions in real-life settings, accounting for environmental and social factors that may enhance or hinder functioning.

Understanding the distinction between capacity and performance is essential to contextualize individual functioning. For instance, an older adult may demonstrate the capacity to walk 500 meters independently in a controlled clinical setting, yet in daily life may only walk short distances indoors due to environmental barriers or fear of falling. Similarly, an individual with mild cognitive impairment may be able to prepare a simple meal when guided step-by-step, but may avoid cooking at home due to safety concerns or lack of social support. These examples highlight the importance of assessments that consider both ideal and real-world functioning, thereby supporting the development of effective, person-centered interventions.

In this study, the percentages of activity limitations and participation restrictions derived from the ICF-C were translated into capacity and performance (CP) qualifiers within the ICF framework. This approach enabled the identification of domains most impacted by disability, as well as areas with the greatest discrepancies between capacity and performance. Analyzing both the concordance and the magnitude of differences between these qualifiers is essential for guiding rehabilitation priorities, evaluating intervention outcomes, and informing the design of integrated health and social care services.

The primary objectives of this study are to (1) quantify the prevalence of disability and cognitive impairment in the study population, (2) assess the reliability and clinical relevance of the capacity and performance qualifiers in the activity and participation domains of the ICF-C, and (3) analyze the concordance and differences between these qualifiers to identify key areas for targeted intervention.

### 1.2. Study purpose

This study aims to analyze measures of capacity and performance to determine whether discrepancies between them serve to restrict or facilitate participation. Limitations in the execution of daily activities can have a profound impact on quality of life. By examining the divergence between capacity and performance qualifier scores, this research seeks to identify the ICF codes most sensitive to these variations and to determine priority areas for intervention. These areas include rehabilitation planning, the evaluation of assistive technology needs, the identification of architectural and environmental barriers, estimation of assistance requirements, and other relevant factors influencing functional outcomes.

## 2. Materials and methods

### 2.1. Study design and sample

Participants were selected from the Social Security registry of individuals aged 50 years and older residing in two distinct geographic areas within the Autonomous Region of Aragón, Spain: a rural area (Cinco Villas) and an urban area comprising two health districts in the city of Zaragoza. Written informed consent was obtained from all participants, or from a legally authorized proxy in cases where the participant was unable to provide consent independently. Recruitment through the Social Security network offered several methodological advantages, including: (1) access to verified medical diagnoses, and (2) enhanced external validity due to the broad population coverage. Exclusions from the initial sample were applied for the following reasons: (1) nonresidence in the study area (317 from Cinco Villas, 15 from Zaragoza), (2) inability to locate the participant (222 from Cinco Villas, 5 from Zaragoza), (3) participant deceased (101 from Cinco Villas, 1 from Zaragoza), and (4) refusal to participate or inability to schedule or attend evaluation appointments (110 from Cinco Villas, 330 from Zaragoza). Additionally, 48 individuals from Cinco Villas were excluded due to incomplete data, resulting in a final sample of 1707 participants with complete functional assessments: 1202 from Cinco Villas and 505 from Zaragoza. Data collection was carried out over approximately three years, encompassing all phases of fieldwork and assessment procedures.

### 2.2. Procedure

The assessment was conducted by five trained interviewers, all with backgrounds in health professions, including two occupational therapists. The evaluation process was organized sequentially into two phases: phase 1–screening and phase 2–diagnostic and functional assessment.

Data collection followed a structured screening protocol conducted in two stages. Sociodemographic variables (e.g., including sex, age, marital status, living arrangements, and education) as well as cognitive status, were collected for the entire sample.

Disability screening was performed using the 12-item version of the World Health Organization Disability Assessment Schedule 2.0 (WHODAS-12), with a positive screen defined as at least one affirmative response.

Cognitive status was assessed with the mini-examen cognoscitivo (MEC), the Spanish adaptation of the mini-mental state examination (MMSE). Participants scoring below 24 (out of 35 points) on the MEC were also classified as screen-positive and proceeded to the full evaluation phase.

Given the large initial sample size in phase 1 (n = 1707), which focused on screening for disability and cognitive impairment, a full diagnostic and functional assessment was conducted in a smaller, randomly selected subsample (n = 129) during phase 2. This two-phase approach was both methodologically sound and logistically feasible, enabling the implementation of resource-intensive assessments while maintaining population representativeness. Random selection of the subsample minimized selection bias and ensured that the in-depth evaluations were generalizable to the broader screened population, thereby enhancing both the internal and external validity of the study findings.

### 2.3. Instrumentation

Disability was assessed using the 12-item version of the WHODAS-12, a standardized instrument that evaluates functional impairment across six domains over the 30 days preceding the assessment: mobility, self-care, getting along with others, life activities, understanding and communication, and participation. Each item is rated on a 5-point Likert scale, ranging from 0 (no difficulty) to 4 (extreme difficulty or inability to perform the activity). Total scores are interpreted using the following severity categories: 1–no problem (0%–4%), 2–mild problem (5%–24%), 3–moderate problem (25%–49%), 4–severe problem (50%–95%), and 5–extreme or complete problem (95%–100%). The WHODAS-12 has been validated across diverse populations and offers a reliable and efficient measure of disability severity 27.

Cognitive function was assessed using the MEC, a widely used screening instrument for cognitive impairment in Spanish-speaking populations. The MEC is a brief cognitive test adapted by Lobo et al. from the original MMSE. A cut-off score of 23 or below is commonly used to indicate the presence of cognitive impairment 28.

The ICF checklist version 2.1A was used in this study 5. This semi-structured tool is designed to elicit and record information on an individual's functioning and disability based on the core categories of the WHO's ICF. The nine chapters from the activities and participation (d) domain included were: d1 “Learning and applying knowledge” (6 codes); d2 “General tasks and demands” (3 codes); d3 “Communication” (5 codes); d4 “Mobility” (9 codes); d5 “Self-care” (11 codes); d6 “Domestic life” (4 codes); d7 “Interpersonal interactions and relationships” (7 codes); d8 “Major life areas” (6 codes); and d9 “Community, social and civic life” (5 codes). A modified version of the ICF-C was developed to enhance its contextual relevance for rural and urban populations aged 50 years and older. This adaptation was informed by expert consultations and conducted in agreement with WHO collaborating groups, with the goal of identifying the most relevant codes for this demographic. The standard ICF scoring system was applied: 0 (no problem), 1 (mild problem), 2 (moderate problem), 3 (severe problem), and 4 (complete problem). To better suit the target population, the following additional ICF items were included: d4600–moving around within the home; d5300–regulating urination; d5301–regulating defecation; d5408–dressing; and d5702–maintaining one's health. A full account of the adaptation process is available upon request. While nature and location are qualitative qualifiers, other domains such as impairment, capacity, performance, and environmental barriers/facilitators are quantified on a 0–100 scale, reflecting the level of difficulty experienced (0 = none; 100 = complete). One-level differences were set to more objectively quantify the differences between the capacity and the performance. According to the definition of functionality in the ICF terminology [23],[28],[29], it was considered important to describe the absolute and relative frequencies in the levels of the capacity and performance qualifiers of the activities and participation component of the ICF-C and to find the percentage differences. The reliability of the data was enhanced by the triangulation process, in which we considered information from participants and companions together with the clinical history and the objective assessment of the trained interviewers [7],[30],[31].

### 2.4. Data analyses

A descriptive analysis of the sample was conducted using frequencies and percentages, stratified by screening results (positive or negative for disability and/or cognitive impairment). Chi-square (χ²) tests were used to assess statistically significant differences in sociodemographic variables between participants with positive and negative screening outcomes. Concordance between ICF qualifiers across different domains was assessed using Cohen's Kappa coefficient. The strength of agreement was interpreted as follows: (1) poor, from 0.2 to 0.4; (2) moderate, from 0.4 to 0.6; (3) good, from 0.6 to 0.8; (4) very good, from 0.8 to 1.0. Kendall's Correlation Coefficient was also calculated to examine the ordinal association between capacity and performance qualifiers, indicating the degree and direction of their relationship. Statistical significance was set at p < 0.05. All analyses were conducted using PASW Statistics version 18.0.0 (SPSS Inc.).

### 2.5. Ethics approval of research

The study was approved by the Research Ethics Committee of the Community of Aragón (ref. P7_2010) and was conducted in accordance with the ethical principles of the World Medical Association's Declaration of Helsinki. All participants signed an informed consent form, and the necessary measures were taken to guarantee the privacy and confidentiality of their personal data. In addition, all organizations involved in the study signed an agreement and gave their permission to conduct the research.

## 3. Results

The final sample consisted of 1707 participants. The response rate to the global interview was 60%. Table 1 shows the socio-demographic data and general characteristics of the total sample. The distribution between men and women was similar in the three age groups. For the age range 50–64 years the prevalence of disability, according to the criteria based on the present study from MEC and WHODAS-12, is 32.1%. It is observed that there is an association (p < 0.001) between age and positive/negative screening. The percentage of screened positive increases as the age range increases.

The results of the subsample in which a full diagnostic and functional evaluation was performed are shown in Table 2, where the absolute and relative frequencies with respect to the total ICF-Checklist capacity and performance qualifiers, along with their p-value, are presented.

**Table 1.** General characteristics of the sample according to positive and negative screenings.

	**TOTAL** N (% column)*	**Positive screening** **N (% row)†**	**Negative screening** **N (% row)†**	**P_value‡**
**Population**	1707 (100%)	864 (50.6%)	843 (49.4%)	0.628
**Sex**				
Men	740 (43.4%)	301 (40.7%)	439 (59.3%)	**<0.001**
Women	967 (56.6%)	563 (58.2%)	404 (41.8%)	
**Age group (years)**				**<0.001**
50–64	670 (39.3%)	215 (32.1%)	455 (67.9%)	
65–79	689 (40.4%)	360 (52.2%)	329 (47.8%)	
≥80	346 (20.3%)	287 (82.9%)	59 (17.1%)	
**Residence**				**<0.001**
At home	1633 (95.7%)	799 (48.9%)	834 (51.1%)	
Nursing home	74 (4.3%)	65 (87.8%)	9 (12.2%)	
**Area of study**				**0.005**
5 Villas (Rural)	1202 (70.4%)	635 (52.8%)	567 (47.2%)	
Zaragoza (Urban)	505 (29.6%)	229 (45.3%)	276 (54.7%)	
**Maximum level of education**				**<0.001**
Less than a primary school	607 (35.6%)	388 (63.9%)	219 (36.1%)	
Primary school	736 (43.2%)	364 (49.5%)	372 (50.5%)	
Higher than primary school	360 (21.1%)	109 (30.3%)	251 (69.7%)	
**Civil status**				**<0.001**
Married/Couple	1135 (66.6%)	502 (44.2%)	633 (55.8%)	
Divorced/Separated	33 (1.9%)	16 (48.5%)	17 (51.5%)	
Single	189 (11.1%)	90 (47.6%)	99 (52.4%)	
Widowed	346 (20.3%)	255 (73.7%)	91 (26.3%)	

Note: *% column: is the percentage of the column. It represents, for each variable, the percentage of each item over the total of the 1707 participants; **†% row:** is the percentage over the row. It represents the percentage of positive and negative screening, within each variable item; **‡**Significance of comparing variables between positive and negative screening: The Chi-squared test was used except for population, where the Binomial test was used.

**Table 2.** Frequencies and concordance between ICF-C capacity and performance qualifiers by Kappa concordance coefficient and Kendall's rank correlation coefficient.

ICF-C	Capacity	Performance				Total	Kendall* p	Kappa† p
		0–4	5–24	25–49	50–100			
Activities and participation	0–4	43 (33.3%)	8 (6.2%)	0 (0.0%)	0 (0.0%)	51 (39.5%)		
	5–24	2 (1.6%)	50 (38.8%)	13 (10.1%)	1 (0.8%)	66 (51.2%)	0.816	0.649
	25–49	0 (0.0%)	0 (0.0%)	7 (5.4%)	4 (3.1%)	11 (8.5%)	<0.001	<0.001
	50–100	0 (0.0%)	0 (0.0%)	0 (0.0%)	1 (0.8%)	1 (0.8%)		
	Total	45 (34.9%)	58 (45.0%)	20 (15.5%)	6 (4.7%)	129 (100%)		
Learning and applying knowledge	0–4	44 (41.1%)	13 (12.1%)	3 (2.8%)	0 (0.0%)	60 (56.1%)		
	5–24	1 (0.9%)	14 (13.1%)	11 (10.3%)	1 (0.9%)	27 (25.2%)	0.766	0.560
	25–49	0 (0.0%)	0 (0.0%)	14 (13.1%)	2 (1.9%)	16 (15.0%)	<0.001	<0.001
	50–100	0 (0.0%)	0 (0.0%)	0 (0.0%)	4 (3.7%)	4 (3.7%)		
	Total	45 (42.1%)	27 (25.2%)	28 (26.2%)	7 (6.5%)	107 (100%)		
General tasks and demands	0–4	54 (41.9%)	4 (3.1%)	4 (3.1%)	3 (2.3%)	65 (50.4%)		
	5–24	1 (0.8%)	28 (21.7%)	1 (0.8%)	3 (2.3%)	33 (25.6%)	0.784	0.784
	25–49	0 (0.0%)	0 (0.0%)	15 (11.6%)	2 (1.6%)	17 (13.2%)	<0.001	<0.001
	50–100	0 (0.0%)	0 (0.0%)	1 (0.8%)	13 (10.1%)	14 (10.9%)		
	Total	55 (42.6%)	32 (24.8%)	21 (16.3%)	21 (16.3%)	129 (100%)		
Communication	0–4	81 (62.8%)	1 (0.8%)	0 (0.0%)	0 (0.0%)	82 (63.6%)		
	5–24	0 (0.0%)	32 (24.8%)	1 (0.8%)	0 (0.0%)	33 (25.6%)	0.983	0.971
	25–49	0 (0.0%)	0 (0.0%)	8 (6.2%)	0 (0.0%)	8 (6.2%)	<0.001	<0.001
	50–100	0 (0.0%)	0 (0.0%)	0 (0.0%)	6 (4.7%)	6 (4.7%)		
	Total	81 (62.8%)	33 (25.6%)	9 (7.0%)	6 (4.7%)	129 (100%)		
Mobility	0–4	39 (30.2%)	7 (5.4%)	0 (0.0%)	1 (0.8%)	47 (36.4%)		
	5–24	1 (0.8%)	42 (32.6%)	8 (6.2%)	3 (2.3%)	54 (41.9%)	0.816	0.718
	25–49	0 (0.0%)	1 (0.8%)	16 (12.4%)	3 (2.3%)	20 (15.5%)	<0.001	<0.001
	50–100	0 (0.0%)	0 (0.0%)	1 (0.8%)	7 (5.4%)	8 (6.2%)		
	Total	40 (31.0%)	50 (38.8%)	25 (19.4%)	14 (10.9%)	129 (100%)		
Self care	0–4	62 (48.1%)	16 (12.4%)	7 (5.4%)	3 (2.3%)	88 (68.2%)		
	5–24	2 (1.6%)	26 (20.2%)	8 (6.2%)	3 (2.3%)	39 (30.2%)	0.536	0.460
	25–49	0 (0.0%)	0 (0.0%)	2 (1.6%)	0 (0.0%)	2 (1.6%)	<0.001	<0.001
	50–100	0 (0.0%)	0 (0.0%)	0 (0.0%)	0 (0.0%)	0 (0.0%)		
	Total	64 (49.6%)	42 (32.6%)	17 (13.2%)	6 (4.7%)	129 (100%)		
Domestic life	0–4	39 (31.0%)	15 (11.9%)	3 (2.4%)	6 (4.8%)	63 (50.0%)		
	5–24	0 (0.0%)	17 (13.5%)	9 (7.1%)	11 (8.7%)	37 (29.4%)	0.625	0.420
	25–49	0 (0.0%)	1 (0.8%)	8 (6.3%)	7 (5.6%)	16 (12.7%)		
	50–100	0 (0.0%)	1 (0.8%)	0 (0.0%)	9 (7.1%)	10 (7.9%)	<0.001	<0.001
	Total	39 (31.0%)	34 (27.0%)	20 (15.9%)	33 (26.2%)	126 (100%)		
Interpersonal interactions and relationships	0–4	58 (45.0%)	1 (0.8%)	0 (0.0%)	0 (0.0%)	59 (45.7%)		
	5–24	3 (2.3%)	40 (31.0%)	2 (1.6%)	0 (0.0%)	45 (34.9%)	0.897	0.879
	25–49	2 (1.6%)	1 (0.8%)	14 (10.9%)	0 (0.0%)	17 (13.2%)	<0.001	<0.001
	50–100	0 (0.0%)	0 (0.0%)	1 (0.8%)	7 (5.4%)	8 (6.2%)		
	Total	63 (48.8%)	42 (32.6%)	17 (13.2%)	7 (5.4%)	129 (100.0%)		
Major life areas	0–4	76 (61.3%)	1 (0.8%)	5 (4.0%)	8 (6.5%)	90 (72.6%)		
	5–24	1 (0.8%)	16 (12.9%)	1 (0.8%)	0 (0.0%)	18 (14.5%)	0.665	0.670
	25–49	0 (0.0%)	1 (0.8%)	6 (4.8%)	3 (2.4%)	10 (8.1%)	<0.001	<0.001
	50–100	0 (0.0%)	0 (0.0%)	1 (0.8%)	5 (4.0%)	6 (4.8%)		
	Total	77 (62.1%)	18 (14.5%)	13 (10.5%)	16 (12.9%)	124 (100.0%)		
Community, social and civic life	0–4	52 (40.6%)	0 (0.0%)	0 (0.0%)	0 (0.0%)	52 (40.6%)		
	5–24	8 (6.3%)	36 (28.1%)	2 (1.6%)	0 (0.0%)	46 (35.9%)	0.806	0.702
	25–49	3 (2.3%)	5 (3.9%)	13 (10.2%)	6 (4.7%)	27 (21.1%)	<0.001	<0.001
	50–100	0 (0.0%)	1 (0.8%)	0 (0.0%)	2 (1.6%)	3 (2.3%)		
	Total	63 (49.2%)	42 (32.8%)	15 (11.7%)	8 (6.3%)	128 (100.0%)		

Note: In this frequency table, the absolute frequencies and the % of the total are presented. The percentage represents, for each variable, the percentage of each cell (combination of scores) over the total number of individuals. *Kendall's Correlation Coefficient. P = Significance of Kendall's Coefficient. **†**Cohen's Kappa Coefficient of Concordance. P = Kappa Coefficient Significance.

The domains with the highest proportion of individuals without disability—defined as capacity or performance scores between 0 and 4—were communication (62.8%) and major life areas (61.3%). The domains with the highest percentage of mild disability (capacity and performance scores between 5–24) were activities and participation (38.8%), mobility (32.6%), and interpersonal interactions and relationships (31%). The highest percentages of individuals with mild disability (scores between 5 and 24) were observed in the domains of activities and participation (38.8%), mobility (32.6%), and interpersonal interactions and relationships (31.0%). For moderate disability (scores between 25 and 49), the most affected domains were learning and applying knowledge (13.1%), mobility (12.4%), interpersonal interactions and relationships (10.9%), and community, social, and civic life (10.2%). The highest prevalence of severe or complete disability (scores between 50 and 100) was found in the domains of general tasks and demands (10.1%), domestic life (7.1%), mobility (5.4%), and interpersonal interactions and relationships (5.4%).

The domains with the most pronounced discrepancies between capacity and performance ratings in cases of moderate to severe disability (scores between 25–49 and 50–100) were domestic life (21.5%), self-care (16.3%), learning and applying knowledge (14%), activities and participation (10.9%), and mobility (8.6%). In the domestic life domain, 21.5% of individuals demonstrated better capacity than performance. Specifically, 42.1% showed moderate or severe disability in performance, compared to 20.6% in capacity. In the self-care domain, performance ratings exceeded capacity by 13.2% among individuals with moderate disability (25–49). This discrepancy widened by 4.7% in the severe/complete disability group (50–100), where 17.9% showed severe disability in performance compared to only 1.6% in capacity. Overall, 16.3% of participants had better capacity than performance in this domain. In the learning and applying knowledge domain, 18.7% of individuals reported moderate to complete limitations in capacity, compared to 32.7% in performance. The percentage of individuals with moderate disability (scores 25–49) was particularly high in performance (26.2%). Overall, 14.0% of participants showed better capacity than performance in this domain. In the activities and participation domain, the proportion of individuals with moderate to severe disability (scores 25–100) in performance was 20.2%, compared to 9.3% in capacity, indicating that 10.9% of individuals had better capacity than performance. In the mobility domain, moderate to severe performance limitations were present in 30.3% of participants, while capacity limitations were reported by 21.7%, with 8.6% exhibiting better capacity than performance.

Conversely, in certain domains, individuals with moderate to severe disability (scores 25–49 and 50–100) demonstrated better performance than their capacity scores indicated. These domains included interpersonal interactions and relationships (10.5%) and community, social, and civic life (5.4%). In the interpersonal interactions and relationships domain, within major life areas, performance was higher than capacity (23.4% vs. 12.9%), with 10.5% exhibiting better capacity than performance.

In the community, social, and civic life domain, capacity limitations were more frequent than performance limitations (23.4% vs. 18.0%), resulting in a performance deficit of 5.4%. Among individuals with moderate disability (scores 25–49), 21.1% reported capacity limitations compared to 11.7% in performance, reflecting a discrepancy of 9.4%.

The difference in concordance between capacity and performance was statistically significant across all domains (p < 0.001). Very good agreement, as indicated by Cohen's Kappa coefficient, was observed in the domains of communication (κ = 0.971) and interpersonal interactions and relationships (κ = 0.879). The remaining domains showed good concordance, with Kappa values ranging from 0.6 to 0.8, except for self-care, domestic life, and learning and applying knowledge, where concordance was lower (κ < 0.5).

In the self-care domain, the percentage of individuals with concordant ability and performance ratings dropped to 68.8%. Among those with scores higher than 25 in either rating, only 8% showed agreement between the two. In the domestic life domain, concordance between capacity and performance ratings dropped to 57.9%.

Overall, Kendall's coefficients were slightly higher than the corresponding Kappa values, indicating stronger ordinal associations. The highest correlations were found in the same domains with high Kappa values, as well as in mobility and community, social, and civic life, the latter exhibiting a particularly strong correlation. In contrast, moderate correlations were observed in the domains of self-care, domestic life, and major life areas.

## 4. Discussion

In this population-based, door-to-door disability screening study, we assessed the reliability and clinical relevance of the capacity and performance qualifiers across the activity and participation domains of the ICF-C. While the health card registry was assumed to offer a comprehensive and up-to-date sampling frame of the target population, our findings revealed certain limitations in its accuracy. Notably, the registry appeared to be partially outdated, leading to sampling bias—particularly an underrepresentation of non-disabled males aged 50–59 years. This was evidenced by the comparable distribution of positively screened individuals across the 50–59 and 60–69 age groups, which may not accurately reflect the actual prevalence of disability by age. Moreover, the scarcity of clinical studies directly comparing capacity and performance qualifier scores hampers the establishment of standardized cut-off points for identifying functional impairments. This lack of benchmark data presents a challenge for interpreting our findings in relation to existing literature, underscoring the need for further research to define clinically meaningful thresholds and to validate the distinction between capacity and performance in diverse populations.

This study offers important contributions in several key areas. First, it demonstrates the feasibility and effectiveness of implementing a simultaneous dual screening strategy for disability and cognitive impairment, conducted by trained health professionals in community settings. Second, the study benefits from a broad geographical coverage, including both urban and rural populations, thereby enhancing the external validity and representativeness of the findings. Third, the research provides a detailed characterization of disability using the ICF-C, with a particular focus on the activity and participation component. By analyzing the relationship between the capacity and performance qualifiers, the study highlights critical discrepancies between what individuals are able to do under ideal conditions versus what they actually do in their current environments, offering a nuanced perspective that may inform rehabilitation, assistive interventions, and policy development.

A novel finding of our study is the identification of notable discrepancies between capacity and performance qualifiers, particularly within the domains of domestic life and self-care, where differences exceed 15%. However, due to the lack of established cut-off points, it remains unclear whether these differences are clinically significant.

Our findings reveal statistically significant differences between capacity and performance across most activities assessed in older adults [18],[32]. Furthermore, the data indicate that older adults with three or more chronic conditions experience greater difficulties in the components of activities and participation, as well as in body functions and structures [21]. Given that limitations in performing activities of daily living can adversely affect quality of life, maintaining a balance between performance and capacity is crucial for health promotion. The observed disparities in qualifier scores underscore potential targets for rehabilitation interventions, such as the implementation of assistive technologies, elimination of architectural barriers, and provision of personal support [18],[32],[33]. The concordance and linear correlation between raters suggest that performance in one domain influences performance in the other [2],[17],[34],[35].

Our findings are consistent with most of the existing literature, which underscores the importance of rehabilitation services. However, due to the absence of environmental factor assessments in this study, we were unable to identify specific areas requiring improvement [13],[18],[28],[30],[32],[35]. Moreover, the use of capacity and performance qualifiers remains insufficiently explored in the literature, resulting in limited evidence regarding their reliability and clinical applicability. The available data suggest that reproducibility improves when assessments are carried out by experienced interviewers and highlight the need for developing context-appropriate qualifiers tailored to each ICF code [7],[13],[19],[20],[32],[36].

The application of CP qualifiers remains one of the major challenges for researchers. A significant limitation of the ICF-C lies in the lack of consensus regarding its use, prompting some experts to urge the WHO to revise and clarify certain codes 32. The differences observed between capacity and performance qualifiers support the need for further research and the development of targeted occupational therapy interventions. Preventing the decline of body functions and structures may contribute to maintaining or enhancing functional capacity in interaction with environmental factors [4],[37].

The inability to accurately assess disability or difficulty among men in performing household tasks may be attributed to gender-based differences. In Spain, domestic responsibilities have traditionally been assumed by women, particularly within older generations. This cultural norm significantly influences the evaluation of disability in the domain of domestic life. Recognizing disability as the result of an interaction between a person's health condition and contextual environmental factors has shifted both the conceptual framework and the direction of intervention—from focusing on the individual to addressing their environment 38. The substantial time required to complete the ICF-C (ranging from 1.5 to 2 hours) may also contribute to a preference for alternative instruments. As such, we recommend the development of simplified versions to support the planning and delivery of social and rehabilitation services.

Future studies should investigate the interaction between environmental factors and the capacity and performance qualifiers, as well as their influence on individuals' daily functioning. Such an approach would support the design of health interventions that are more precisely tailored to individual needs.

## 5. Methodological considerations/limitations

This study is pioneering in establishing a basis for the differences that can be found between capacity and performance qualifiers in people aged 50 years and over.

However, several limitations should be considered: the study's cross-sectional design prevents us from inferring causality or evaluating the directionality of associations. The entire population belonged to the same region, so extrapolation of the results to the general population should be done with caution. Participation was conditioned by the participants' work activities, which may have influenced the actual detection of screening. The limitations of men's domestic responsibilities in traditionally female-headed family units were overlooked in the counts.

## 6. Conclusions

This study reveals a high prevalence of disability among adults aged 50 and older, with significant functional limitations observed in the domains of general tasks, domestic life, and self-care. Marked discrepancies between capacity and performance—particularly in domestic life and learning—point to the presence of environmental barriers that constrain individuals' ability to function optimally. Conversely, domains such as community life and interpersonal relationships, where performance exceeds capacity, underscore the enabling influence of supportive social and environmental contexts. These findings offer a nuanced understanding of the gap between potential and actual functioning in daily life. Recognizing this gap is essential for identifying opportunities to enhance functional ability and for addressing both environmental facilitators and barriers. The results support the clinical utility of the ICF-C and emphasize the importance of incorporating environmental factors into disability management strategies. Such an approach enables healthcare professionals to develop targeted interventions, optimize resource allocation, and ultimately improve autonomy and quality of life in ageing populations.

## Use of AI tools declaration

The authors have used AI (ChatGPT) in this work to help edit or summarize some paragraphs of the text, correct or identify errors in English grammar or syntax. The authors have taken responsibility for eliminating possible biases in the text due to the AI.
